# Developing a *Chromochloris zofingiensis* Mutant for Enhanced Production of Lutein under CO_2_ Aeration

**DOI:** 10.3390/md20030194

**Published:** 2022-03-07

**Authors:** Yuanyuan Ren, Jinquan Deng, Yan Lin, Junchao Huang, Feng Chen

**Affiliations:** 1 Institute for Food and Bioresource Engineering, College of Engineering, Peking University, Beijing 100871, China; 1701111648@pku.edu.cn; 2 Shenzhen Key Laboratory of Marine Microbiome Engineering, Institute for Advanced Study, Shenzhen University, Shenzhen 518060, China; 1900392002@email.szu.edu.cn (J.D.); yanhely@163.com (Y.L.); 3 Institute for Innovative Development of Food Industry, Shenzhen University, Shenzhen 518060, China

**Keywords:** microalgae, *Chromochloris zofingiensis*, lutein, CO_2_ aeration, cGMP-dependent kinase

## Abstract

Microalgae are competitive and commercial sources for health-benefit carotenoids. In this study, a *Chromochloris zofingiensis* mutant (*Cz-pkg*), which does not shut off its photosystem and stays green upon glucose treatment, was generated and characterized. *Cz-pkg* was developed by treating the algal cells with a chemical mutagen as *N*-methyl-*N’*-nitro-*N*-nitrosoguanidine and followed by a color-based colony screening approach. *Cz-pkg* was found to contain a dysfunctional cGMP-dependent protein kinase (PKG). By cultivated with CO_2_ aeration under mixotrophy, the mutant accumulated lutein up to 31.93 ± 1.91 mg L^−1^ with a productivity of 10.57 ± 0.73 mg L^−1^ day^−1^, which were about 2.5- and 8.5-fold of its mother strain. Besides, the lutein content of *Cz-pkg* could reach 7.73 ± 0.52 mg g^−1^ of dry weight, which is much higher than that of marigold flower, the most common commercial source of lutein. Transcriptomic analysis revealed that in the mutant *Cz-pkg*, most of the genes involved in the biosynthesis of lutein and chlorophylls were not down-regulated upon glucose addition, suggesting that PKG may regulate the metabolisms of photosynthetic pigments. This study demonstrated that *Cz*-*pkg* could serve as a promising strain for both lutein production and glucose sensing study.

## 1. Introduction

Lutein is a natural carotenoid that has drawn great interest for its health-promoting functions, such as scavenging free radicals, preventing age-related macular degeneration (AMD) and Alzheimer’s Disease (AD), and beneficial for skin health [1,2]. At present, commercial lutein mostly derives from marigold petals, while harvesting only in specific seasons coupled with time-consuming petal collection hinders the large-scale production [3]. Though lutein is also common in vegetables, its daily dietary uptake is still insufficient for all populations. Thus, searching for better lutein sources as nutritional supplement is of significance. 

Microalgae are potent sources of carotenoids that served as either primary carotenoids for photosynthesis or secondary ones in response to adverse conditions [4]. Serving as an essential photosynthesis pigment, the production of lutein in microalgae is related to photosynthetic activity. Compared with terrestrial plants, microalgae have higher photosynthetic efficiency and growth rates [4]. A number of microalgal species, including *Chlorella protothecoides* [5], *Parachlorella* sp. JD-076 [6], *Scenedesmus* sp. [7], and *Chlorella vulgaris* UTEX 265 [8], have been investigated for lutein production with limiting successes. 

*Chromochloris zofingiensis* is a green microalga in the class *Chlorophyceae* that can grow fast under autotrophy, mixotrophy, and heterotrophy [9]. *C. zofingiensis* switched off photosynthesis in the presence of glucose, resulting in degrading of chlorophylls and accumulation of the secondary astaxanthin [10]. We had previously characterized a *Cz-bkt1* mutant that failed to accumulate astaxanthin, but instead accumulated high amounts of zeaxanthin when induced with high light and glucose [11]. 

In this study, we developed and characterized a novel mutant strain of *C. zofingiensis* that did not shut off its photosynthetic system under mixotrophic cultivation, and therefore could maintain high photosynthetic activity, cell growth and accumulated much higher amounts of pigments including lutein under various culture conditions. The objectives of this study are (1) to uncover the mutated gene and explain the phenotype differences between the mutant and the wild type; (2) to investigate the transcriptome differences between the mutant and the wild type; (3) to find the optimal CO_2_ concentration for high yield and productivity of lutein from the mutant; and (4) to put forward a novel hypothesis of glucose-sensing in microalgae.

## 2. Results

### 2.1. Isolation of a “Stay-Green” Mutant of C. zofingiensis

MNNG (*N*-methyl-*N’*-nitro-*N*-nitrosoguanidine) has been proved to be an effective chemical for creating microalgal mutants with enhanced production of carotenoids [11]. In this study, MNNG was applied to generate mutants of *C. zofingiensis* followed by growing the treated cells on plates with Kuhl medium containing 15 g L^−1^ for three weeks. Generally, red colonies appeared due to the accumulation of red ketocarotenoids in the algal cells induced by glucose [12]. However, we found a green colony (here we named *Cz-pkg*) that might fail to accumulate ketocarotenoids whereas maintain stable photosynthesis pigments. 

**Figure 1:**
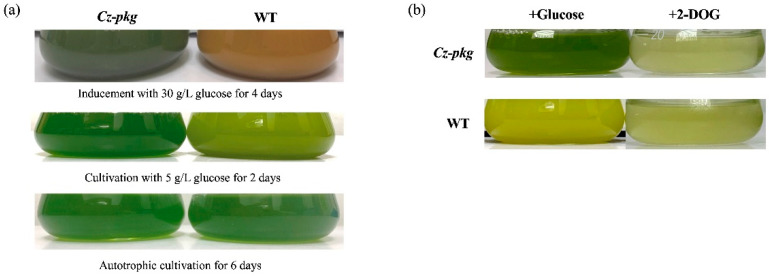
(**a**) Phenotypes of *Cz-pkg* and WT cultures under autotrophy, 5 g L^−1^ and 30 g L^−1^ glucose-addition mixotrophy. (**b**) Growth status of *Cz-pkg* and WT cultures under 5 g L^−1^ glucose or 2-DOG cultivation on Day 3.

To characterize this apparent difference, the *Cz-pkg* was picked out and went on cultivation in liquid Kuhl medium with or without glucose. When cultivated in medium without glucose, both *Cz-pkg* and WT (wild type) cell cultures appeared green (Figure 1a). However, in the culture of medium containing glucose, *Cz-pkg* displayed green color while WT showed yellow to orange color (Figure 1a).

**Figure 2:**
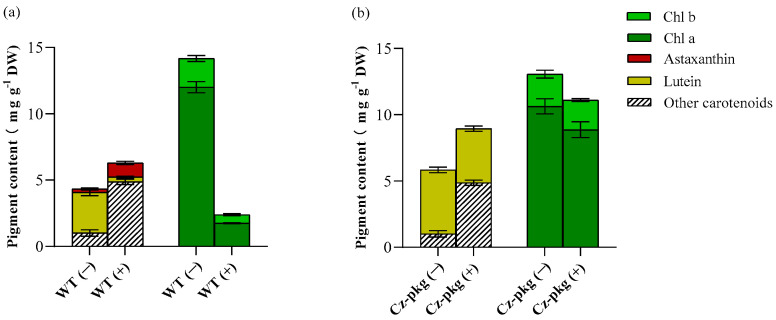
Pigment profiles of (**a**) WT and (**b**) *Cz-pkg* under autotrophy or glucose inducement on Day 6. (−) and (+) represent cultures without glucose or with 30 g L^−1^ addition. Data in the figure were presented in the form of means (*n* = 3) ± the standard deviation.

HPLC analysis showed that under photoautotrophic condition, both WT and *Cz-pkg* shared similar pigment profiles under autotrophy, mainly as chlorophyll a, chlorophyll b, and lutein (Figure 2). However, when induced by 30 g L^−1^ glucose, WT accumulated mostly ketocarotenoids, mainly astaxanthin (1.06 ± 0.12 mg g^−1^ DW) (Figure 2). In contrast, *Cz*-*pkg* mainly accumulated chlorophylls and lutein (4.08 ± 0.19 mg g^−1^ DW), which was over 10-fold of WT (0.37 ± 0.06 mg g^−1^ DW). Thus, *Cz-pkg* might be a potential strain for lutein accumulation and production under various culture conditions.

### 2.2. A Nonsense Mutation Occurred in PKG Gene of Cz-pkg

Since glucose failed to shut off the photosynthesis of *Cz-pkg* nor induce astaxanthin production, it is possible that a very regulating gene may loss its function. Hexokinase (HXK) is a conserved enzyme in generating glucose-6-phosphate from glucose in sugar metabolism. This reacting step was revealed to involve in switching off the photosynthesis of *C. zofingiensis* [10]. Thus, we first proposed that our stay-green *Cz-pkg* might loss its HXK function. *C. zofingiensis* consists of only one *HXK* gene [10], and we cloned and sequenced the *HXK* gene of *Cz-pkg*; however, no difference was found in the genes between WT and the mutant (data not shown).

2-DOG is a glucose analog commonly used to investigate sugar sensing in cells. 2-DOG can be uptake into microalgal cells and phosphorylated by hexokinase, however, the product cannot be further metabolized [10]. When treated with 2-DOG, both WT and *Cz-pkg* died due to the photosynthetic switching off (Figure 1b), supporting that HXK was normal in *Cz-pkg*. For a comprehensive knowledge of the mutation in *Cz-pkg*, transcriptome analysis was applied to find out the possible mutation point.

As proposed, we focused on nonsense mutation in regulation genes. Single-nucleotide polymorphisms (SNP) analysis located a SNP occurred in a cGMP-dependent protein kinase (PKG) gene that an A to T substitution led to the change of TTG (encoding for leucine) to UAG (stop codon), resulting in nonsense mutation of the PKG gene (Figure S1). PKG plays an essential role in sensing guanosine-3′, 5′-cyclic monophosphate (cGMP) in diverse physiological processes in animals and plants in the NO-cGMP-PKG pathway [13,14]. Moreover, we determined the transcriptional levels of the key genes involved in the biosynthesis of photosynthetic pigments.

### 2.3. Glucose Differentially Regulates the Biosynthesis of Photosynthetic Pigments

**Figure 3:**
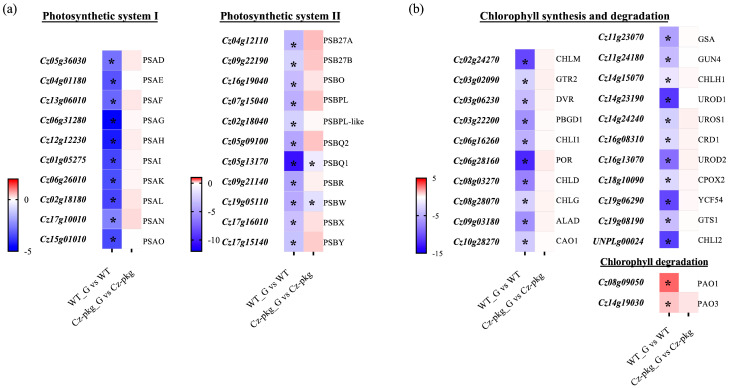
Different expressions involved in photosynthesis between WT and *Cz-pkg* mixotrophic cells with 30 g L^−1^ glucose addition. Heat map illustrating differences of mRNA levels of the genes related with (**a**) photosystem, (**b**) chlorophyll synthesis and degradation. WT_G and Cz-pkg_G represent cultures with 30 g L^−1^ glucose. Fold change of the mRNA levels was calculated as Log_2_FC and displayed in the heat map. Significant difference (at least a two-fold change and FDR adjusted *p* < 0.05) is indicated with an asterisk. PSA, Photosystem I Subunit; PSB, Photosystem II Subunit; CHLM, Mg-Protoporphyrin IX Methyltransferase; GTR2, Glutamyl tRNA Reductase; DVR, Divinyl Chlorophyllide a 8-Vinyl Reductase; PBGD1, Porphobilinogen Deaminase; CHLI1, Mg-Chelatase Subunit I; POR, Light-Dependent Protochlorophyllide Oxidoreductase; CHLD, Mg-Chelatase Subunit D; CHLG, Chlorophyll Synthetase; ALAD, Delta-Aminolaevulinic Acid Dehydratase; CAO1, Chlorophyllide a Oxygenase; GSA, Glutamate-Semialdehyde Aminotransferase; GUN4, Tetrapyrrole Binding Protein; CHLH1, Mg-Chelatase Subunit H; UROD1, Uroporphyrinogen III Decarboxylase; UROS1, Uroporphyrinogen III Synthase; CRD1, Mg-Protoporphyrin Monomethyl Ester Cyclase; UROD2, Uroporphyrinogen III Decarboxylase; CPOX2, Coproporphyrinogen-III Oxidase; YCF54, Ycf54 Conserved Hypothetical Protein; GTS1, Glutamyl-Glutaminyl Non-Discriminatory tRNA Synthetase; CHLI2, Mg-Chelatase Subunit I; PAO1, Pheophorbide a Oxygenase; PAO3, Pheophorbide a Oxygenase.

qRT-PCR was used to detect the transcription of genes encoding for the components of photosystem II (PS II) and photosystem I (PS I), which participate in the initial steps of photosynthesis, driving solar energy into chemical energy for the biosynthesis of organic compounds in oxygenic photosynthetic organisms [15]. As shown in Figure 3a, most genes of photosystem I and II were significantly downregulated in WT under 30 g L^−1^ glucose inducement, consistent with the shut off photosynthesis (Figure 1a). In contrast, except for PSBQ1 and PSBW of photosystem II, most of the genes were slightly upregulated in *Cz-pkg*. In addition, most genes involved in chlorophyll formation were significantly downregulated in WT, while there were no significant changes in *Cz-pkg* when induced with glucose (Figure 3b), some were even slightly upregulated. Typically, light-dependent protochlorophyllide oxidoreductase (POR), a key enzyme in chlorophyll synthesis, responsible for the successive reduction to form chlorophyllide a [16], was significantly downregulated in WT (12.14-fold decrease). Similarly, Mg-protoporphyrin IX methyltransferase (CHLM) was also significantly downregulated in WT (over 10-fold decrease) (Figure 3b). 

Pheophorbide a oxygenase (PAO) is a key enzyme for chlorophyll degradation [17]. As shown in Figure 3b, under 30 g/L glucose inducement, apart from downregulation of chlorophyll synthesis, WT cells went through upregulation of chlorophyll degradation, leading to the orange color of its suspension culture (Figure 1). In contrast, *Cz-pkg* exhibited stable expression of the related genes (Figure 3) and maintained stable contents of chlorophylls (Figure 2) under 30 g L^−1^ glucose induction. As a result, *Cz-pkg* displayed stay-green phenotype (Figure 1) and contained much higher amounts of lutein than WT (Figure 2).

**Figure 4:**
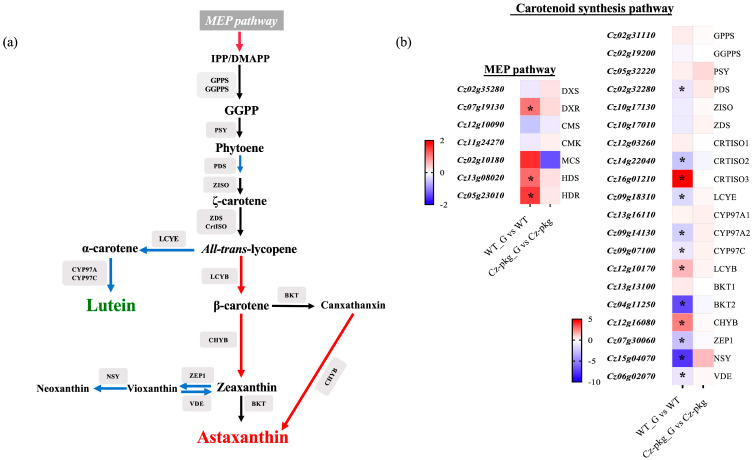
(**a**) Carotenoid biosynthetic pathways in *C. zofingiensis*. Red arrows indicate pathways with significant up-regulations, and blue arrows indicate pathways with significant down-regulations. (**b**) Heat map illustrating differences of gene expressions related with carotenogenesis between WT and *Cz-pkg* under 30 g L^−1^ glucose. Fold change of the mRNA levels was calculated as Log_2_FC and displayed in the heat map. Significant difference (at least a two-fold change and FDR adjusted *p* < 0.05) is indicated with an asterisk. DXS, 1-deoxy-d-xylulose 5-phosphate synthase; DXR, 1-deoxy-d-xylulose 5-phosphate reductoisomerase; CMS, 2-C-methyl-d-erythritol 4-phosphate cytidylyltransferase; CMK, 2-C-methyl-d-erythritol 4-phosphate cytidylyltransferase; MCS, 2-C-methyl-d-erythritol 2,4-cyclodiphosphate synthase; HDS, 4-hydroxy-3-methylbut-2-en-1-yl diphosphate synthase; HDR, 4-hydroxy-3-methylbut-2-en-1-yl diphosphate reductase; GPPS, geranyl diphosphate synthase; GGPPS, geranylgeranyl pyrophosphate synthase; PSY, Phytoene Synthase; PDS, Phytoene Desaturase; ZISO, Zeta-Carotene Isomerase; ZDS, Zeta-Carotene Desaturase; CRTISO, Carotene Isomerase; LCYE, Lycopene Epsilon-Cyclase; CYP97, Cytochrome P450-Type Carotene Hydroxylase; LCYB, Lycopene Beta-Cyclase; BKT, Beta-Ketolase; CHYB, Beta-Carotene Hydroxylase; ZEP1, Zeaxanthin Epoxidase; NSY, Neoxanthin Synthase (ABA4); VDE, Violaxanthin de-epoxidase.

To further understand the different regulation of carotenoid biosynthesis between WT and *Cz-pkg*, we detected the expression of genes related to carotenoid biosynthesis (Figure 4). Under 30 g L^−1^ glucose, the expression of essential enzymes involved in the MEP (methylerythritol phosphate) pathway was significantly upregulated in WT, such as DXR (1-deoxy-d-xylulose 5-phosphate reductoisomerase), HDS (4-hydroxy-3-methylbut-2-en-1-yl diphosphate synthase) and HDR (4-hydroxy-3-methylbut-2-en-1-yl diphosphate reductase). Moreover, the expression of LCYB (Lycopene Beta-Cyclase) to the β-branch carotenoids was significantly upregulated, leading to more isoprenoid skeletons for astaxanthin and canthaxanthin; while the expression of enzymes leading to lutein were significantly downregulated, such as LCYE (Lycopene Epsilon-Cyclase), CYP97A2 (Cytochrome P450-Type Carotene Hydroxylase), and CYP97C. In addition, the downregulated expression of ZEP 1 (Zeaxanthin Epoxidase) and NSY (Neoxanthin Synthase) restricted the formation of vioxanthin and neoxanthin, leading to more zeaxanthin for astaxanthin synthesis in WT. In contrast, there are no significant expression changes in *Cz-pkg*. Thus, *Cz-pkg* could have a potential for sustainable production of lutein.

### 2.4. The Growth of Cz-pkg under Different Trophic Modes

To assess if *Cz-pkg* has potential for lutein production, mixotrophic cultivation with gradient concentrations of glucose (from 5 g L^−1^ to 50 g L^−1^) was applied to find out the best growing condition. Though high glucose concentration increased biomass with longer cultivation time, the maximum specific growth rates showed a descending trend with the increase of sugar concentration, and it was the highest at 5 g L^−1^ (*u*_max_= 0.0375 ± 0.0025 h^−1^, Figure S2). This result is consistent with previous research that 5 g L^−1^ glucose addition is the optimal condition for cultivation of *C. zofingiensis*. [18]. Thus, for mixotrophy of *Cz-pkg*, 5 g L^−1^ was chosen as the optimal glucose concentration for further experiments.

**Figure 5:**
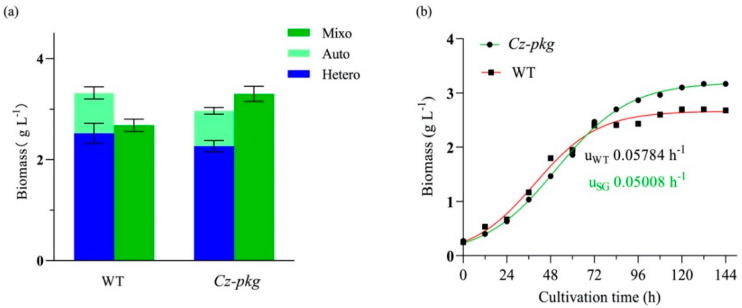
(**a**) Biomass of WT and *Cz*-*pkg* under different trophic modes on Day 5 in flasks; (**b**) growth curve of WT and *Cz*-*pkg* under 5 g L^−1^ glucose in flasks. Solid symbols represent actual sampling points, while red and green curves as fitted growth curves (R^2^ > 0.99). Data were presented in the form of mean ± the standard deviation (*n* = 3).

The biomass concentration of *Cz-pkg* under mixotrophy was higher than the sum of the concentrations under autotrophy and heterotrophy (Figure 5a). In contrast, as WT shut off photosynthesis in the presence of glucose, its biomass under mixotrophy was lower than the sum of those under autotrophy and heterotrophy (Figure 5a). As shown in Figure 5b, the growth curves of *Cz-pkg* and WT under 5 g L^−1^ glucose were determined and fitted to a logistic growth model by Prism (R^2^ > 95%). According to the fitted results, *Cz-pkg* could accumulate 1.23-fold biomass of WT, although it reached the plateau stage later and its fit-calculated maximum specific growth rate (*u*_max_ = 0.05008 h^−1^) was lower than that of WT (*u*_max_ = 0.05784 h^−1^). The slow growth rate may be due to its slower utilization of glucose. As *Cz-pkg* keeps green in the presence of glucose, its photosystem can work efficiently for uptaking both inorganic (CO_2_) and organic carbon sources (glucose) under mixotrophic cultivation. 

### 2.5. Supplemented CO_2_ Promotes Cell Growth and Lutein Production

In contrast to marigold flower that accumulates esterified lutein, microalgae produce lutein mostly in free form [1]. Up until now, only a limited number of microalgae have been exploited for lutein production, and typically their lutein contents range about 340 to 760 mg/100 g DW [19]. CO_2_ was shown to increase algal photosynthesis [20]. Since *Cz-pkg* does not shut off photosynthesis in the presence of glucose, five cultivation conditions (flasks without aeration, air, 2.5% CO_2_, 4% CO_2_, and 5% CO_2_) were performed under both autotrophy and mixotrophy to evaluate the effects of trophic mode and CO_2_ concentrations on cell growth and lutein accumulation. 

**Figure 6:**
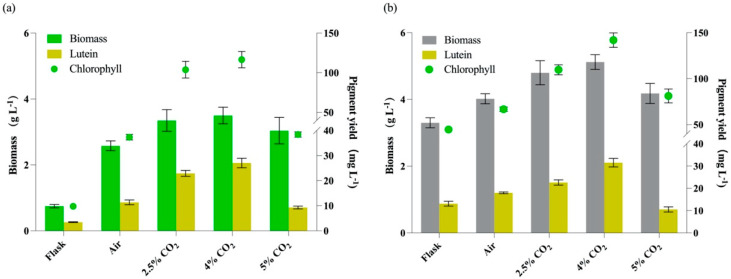
The growth and pigment production of *Cz*-*pkg* under different culture conditions. (**a**) Autotrophic cultures in flasks or aerations with different CO_2_ concentrations. (**b**) Mixotrophic cultures with 5 g L^−1^ glucose addition in flasks or aerations with different CO_2_ concentrations. Data were presented in the form of mean ± the standard deviation (*n* = 3).

As shown in Figure 6, all cultures with aeration showed higher biomass than those in flasks, whether under autotrophy or mixotrophy, which may be due to better gas exchange under aeration and light exposure of cells. However, when the CO_2_ concentration reached 5%, the biomass concentrations and lutein yields showed reverse tendency. 

As shown in Table 1, 5% CO_2_ led to a decrease in the content of photosynthetic pigments (both chlorophylls and lutein), and the proportion of lutein under mixotrophy with 5% CO_2_ decreased to 44.4% of total carotenoids, nearly two-fold lower than other cultures. HPLC analysis revealed that *Cz-pkg* also accumulated zeaxanthin up to 1.70 mg g^−1^ DW, which occupied about 30% of the total carotenoids. Higher concentrations of CO_2_ had previously been found to cause lower photosynthetic efficiency and cell growth of *Chlorella minutissima* [21] and *Desmodesmus* sp. [22], possibly resulting from excessive changes of pH values in the medium led by high soluble CO_2_ concentrations. 

As shown in Figure 6a, 4% CO_2_ under autotrophy showed the best on both biomass and lutein accumulation, and the lutein yield under autotrophy can reach 27.83 ± 1.87 mg L^−1^, which was 8.02-fold of that in flask and 2.46-fold of that with air aeration (0.04% CO_2_). As shown in Figure 6b, under mixotrophy, the lutein yields were relatively higher than those with the same CO_2_ aeration under autotrophy. This result is consistent with a previous study that mixotrophy was more favorable for lutein production [6]. The maximal lutein yield under mixotrophy was 31.93 ± 1.91 mg L^−1^ with 4% CO_2_, which was 2.43-fold of that in flask and 1.78-fold of that with air aeration. Besides, the content of lutein was in positive correlation with the content of chlorophylls (Figure 6). 

To further find out the pattern of lutein accumulation in *Cz-pkg*, we determined the time-course lutein content during cultivation. As shown in Table 2, lutein began to increase rapidly when the cells entered the plateau phase (from Day 6 to Day 8), which may be due to the increased cell density, so more light-harvesting pigments are needed to meet the energy required for cell growth. Yeh et al. [23] suggested that in the later growth stage of *Desmodesmus* sp., lutein accumulation was essential for maintaining the structural integrity of LHCs and promoted photosynthesis under low light conditions due to the self-shading effect. 

At present, marigold flower is still the major source of natural lutein, and it contains lutein from 0.17 to 5.70 mg g^−1^ [3]. Compared with marigold, the concentrations of lutein in microalgae are much higher [19]. As most microalgae contain lutein less than 5 mg g^−1^ DW [11], microalgal species containing lutein more than 5 mg g^−1^ have been acknowledged as potential sources for lutein production [19].

With 4% CO_2_ aeration under mixotrophy, *Cz-pkg* can achieve 6.28 ± 0.57 mg g^−1^ DW of lutein with a yield of 31.93 ± 1.91 mg L^−1^. Under autotrophy, *Cz-pkg* can also accumulate large content of lutein as 7.73 ± 0.52 mg g^−1^ DW with a relatively high yield of 27.83 ± 1.87 mg L^−1^. As shown in Table 2, the maximal productivity of lutein was achieved also under mixotrophy with 4.0% CO_2_ as 10.57 ± 0.73 mg L^−1^ day^−1^, 1.20-fold of that under autotrophy (8.80 ± 0.60 mg L^−1^ day^−1^), which is the highest among the known species for lutein production as listed in Table 3. Considering that the production of carotenoids by *C. zofingiensis* can be greatly enhanced by optimization of culture conditions as reported by previous studies [11,19,24], *Cz-pkg* may serve as a potential source for lutein production on industrial scales.

## 3. Discussion

As sugars play vital roles in regulations of cell growth, physiology, metabolism, and gene expression in plants and microalgae, it is of great significance to investigate the genes and enzymes associated with glucose sensing and responding. Roth et al. previously found that glucose-treated *hxk1* mutants did not shut off photosynthesis or accumulate astaxanthin in the presence of glucose under light, same as *Cz-pkg* in this research [10]. They hypothesized that HXK was necessary for glucose-mediated photosynthesis repression, and G-6-P (glucose-6-phosphate), the downstream product of glucose phosphorylated by hexokinase (HXK) was also closely related [10]. Though the functions of PKG have been shown important in plants, its definite properties and functions still remain unclear [26,27]. As *PKG* mutant *Cz-pkg* does not shut off photosynthesis or accumulate astaxanthin in the presence of glucose, the kinase PKG might be significant in regulating downstream metabolism after microalgae sensing glucose. Further study should focus on exploiting the functions of PKG and determine its role in glucose sensing of microalgae with molecular methods, such as *CRISPR/Cas9*, to achieve rigor results.

## 4. Materials and Methods

### 4.1. Microalgae Strain and Cultivation

*C. zofingiensis* (ATCC 30412) was obtained from American Type Culture Collection (ATCC, Rockville, MD, USA) and cultivated in Kuhl medium as reported previously [11]. The Kuhl medium contains KNO_3_ 1.01 g L^−1^, NaH_2_PO_4_·H_2_O 0.62 g L^−1^, Na_2_HPO_4_·2H_2_O 0.089 g L^−1^, MgSO_4_·7H_2_O 0.247 g L^−1^, CaCl_2_·2H_2_O 14.7 mg L^−1^, Na_2_EDTA·H_2_O 6.95 mg L^−1^, FeSO_4_·7H_2_O 6.95 mg L^−1^, H_3_BO_3_ 0.061 mg L^−1^, MnSO_4_·H_2_O 0.169 mg L^−1^, ZnSO_4_·7H_2_O 0.287 mg L^−1^, CuSO_4_·5H_2_O 0.0025 mg L^−1^, (NH_4_)_6_Mo_7_O_24_·4H_2_O 0.01235 mg L^−1^. Briefly, the algal cells were cultured in 100 mL fresh Kuhl medium containing 5 g L^−1^ glucose (in 250-mL flasks) with orbital shaking at 150 rpm, and illuminated with continuous light of 30 μmol photons m^−2^ s^−1^ (cool-white fluorescent tube light). Cells grown to late exponential phase were used as seed cells for further experiments. For cultivation in bubble tubes, the seed cells were inoculated into glass columns at 0.5 g L^−1^ under illumination of 150 μmol photons m^−2^ s^−1^ from one side, and separately aerated by air, 2.5% (*v*/*v*), 4% (*v*/*v*), and 5% (*v*/*v*) CO_2_-enriched air in different experimental groups. All experiments were operated in triplicate. Cell biomass was determined according to our previous study [28]. By sampling at a 12 h time interval, the biomass concentrations were fitted according to a logistic growth model as the following equation:

(1) $${Y=Y}_{M}{\times Y}_{0}/\left(\left(Y_{M}-Y_{0}\right){\times e}^{\left(-\mathrm{kx}\right)}{+Y}_{0}\right)$$

where Y_M_ is the maximum biomass (g L^−1^), Y_0_ is the initial inoculum biomass (g L^−1^), k is the rate constant (h^−1^), and x is the cultivation time (h).

### 4.2. Mutant Generation, Selection, and Identification

The mutagenesis procedure by *N*-methyl-*N’*-nitro-*N*-nitrosoguanidine (MNNG) was performed successfully according to our previous study [29]. Surviving colonies were then isolated and transferred to fresh 1/2 Kuhl medium with or without 15 g L^−1^ glucose. In detail, the selection procedure was conducted as follows: (a) each of single colonies were suspended in two liquid cultures (with or without glucose); (b) a clone showing green color in the presence of glucose was isolated. The culture of the stay-green mutant and the wild type were then transferred to Kuhl medium with 5 mM 2-deoxy-D-glucose (2-DOG) to identify if the mutant strain shuts off photosynthesis in the presence of glucose according to [10]. To eliminate the mutant with transport deficiency of glucose, the mutant was also cultivated in dark in the presence of glucose.

### 4.3. Pigment Extraction and Analysis

Algal cells were harvested after centrifugation, and the pellets were lyophilized and grinded in 2 mL tubes with 3 stainless steel beads for 10 × 30 s at 1/30 frequency with the TissueLyser II (QIAGEN, Hilden, Germany). Then, the debris were extracted with pre-chilled acetone (HPLC grade) for three times until they were almost colorless. The supernatants were collected by centrifugation (13,000× *g* for 10 min at 4 °C) and filtered through a 0.22 μm Millipore organic membrane. Absorbance values at 470, 652.4, and 665.2 nm were measured with a spectrophotometer for pigment quantifications according to our previous study [28]. Pigment profile analysis was performed by high performance liquid chromatography (HPLC, Waters) according to Huang et al. with modifications [11]. Briefly, HPLC was equipped with a Waters YMC Carotenoid C30 column (5 μm, 4.6 × 250 mm), and the mobile phase consisted of solvent A (methanol: isopropanol, 68:32, *v*/*v*) and solvent B (acetonitrile: methanol: water, 84:2:14, *v*/*v*/*v*). A total of 10 μL of each sample was analyzed at a flow rate of 0.80 mL min^−1^ with a gradient mode (0−15 min: linear gradient of 0−100% of A; 15−30 min: linear gradient of 0−100% of B). Compounds were detected at 450 and 480 nm. The peaks of each compound were identified by their absorption spectra, and the retention times were compared with the biological reference standards for recognition and quantification. The lutein productivity was calculated with the following equation:

(2) $${\mathrm{Productivity}\;(\mathrm{mg}\;L}^{-1}{\mathrm{day}}^{-1})=\frac{{\mathrm{Biomass}\;\mathrm{concentration}\;(g\;L}^{-1}{)\;\times \;\mathrm{Lutein}\;\mathrm{content}\;(\mathrm{mg}\;g}^{-1})}{\mathrm{Cultivation}\;\mathrm{time}\;(\mathrm{day})}.$$

### 4.4. DNA Extraction and Molecular Characterization of Mutant

Genomic DNA of *C. zofingiensis* was extracted with Chelex-100 chelating resin (Bio-Rad Laboratories, Hercules, CA, USA) according to Kang et al. [30]. Briefly, microalgal cells were centrifuged at 14,000 rpm for 1 min, and the supernatant was discarded. Then, 1 mL PBS solution was added, vortexed and centrifuged to wash the cells twice. A total of 100 μL of autoclaved 5% Chelex-100 chelating resin (suspended in 0.1M Tris and 0.5 mM EDTA, pH = 8.0) was added, vortexed and boiled at 100 °C for 20 min. The suspension was then and centrifuged at 14,000 rpm for 1 min. The supernatant was collected and used as DNA samples for the characterization experiments.

To reveal the molecular base of the mutant, DNA sequences of putative mutated genes were compared with that of wild type by PCR amplification and sequencing. According to the whole-genome of *C. zofingiensis* [31], primers were designed to amplify the full length of *CzHXK1* and *CzPKG* genes as follows: HXK1F: 5′ ATGAAACTTGACGCAGACACTCAACG 3′ and HXK1R: 5′ TTAGGCAGTAGTGCTTGGCAGGGGGTC 3′ for HXK1; and PKGF: 5′ ATGGGGAACTCGCACAGCCAG 3′ and PKGR: 5′ TGAGCAGTGATGTAGCACTGGCAG 3′ for PKG. The PCR procedure was set as: 98 °C for 3 min, 36 cycles of 98 °C for 10 s, 60 °C for 5 s, and 68 °C for 6 min (for *CzHXK1*) or 8 min (for *CzPKG*). An elongation procedure was added at 68 °C for 5 min. PCR products were gel purified and sequenced. Sequence alignments were completed through BLAST online (https://blast.ncbi.nlm.nih.gov/Blast.cgi, accessed on 10 October 2021).

### 4.5. Transcriptome Sequencing and Analysis of Differentially Expressed Genes

Algal cultures (both Cz-WT and *Cz-pkg*) in the presence of glucose were harvested at exponential phase for RNA isolation and sequencing with an Illumina Novaseq 6000 system (Illumina Inc., San Diego, CA, USA) by Majorbio Bio-pharm Technology Co., Ltd. (Shanghai, China). The transcriptome sequences are accessible in the NCBI Sequence Read Archive database (http://www.ncbi.nlm.nih.gov/sra/, accessed on 10 October 2021) under the accession number PRJNA664005. Expression of the annotated genes was profiled by the values of TPM (transcripts per million reads) through RSEM v1.3.1. Fold change of a gene between two samples was considered significant when $|\log_{2}\left({\mathrm{TPM}}_{\mathrm{Sample}\;1}{/\mathrm{TPM}}_{\mathrm{Sample}\;2}\right)|\;\geq \;1$ with Padjust < 0.001.

### 4.6. RNA Isolation and Quantitative Real-Time PCR

Validation of the interesting key genes was also performed by quantitative real-time PCR (qRT-PCR) analysis using the primers listed in Table S1. Total RNA was isolated with TRIzol reagent (Invitrogen, Shanghai, China) according to the manufacturer’s protocol. To remove possible contaminating DNA, RNase-free DNase I (TaKaRa, Beijing, China) was used to treat raw RNA samples. Nanodrop 2000 (Thermo Scientific, Shanghai, China) was used to determine the concentration and quality of RNA was checked by electrophoresis. Total RNA (~1 μg) was then reversely transcribed to cDNA using Prime ScriptTM RT reagent kit (TaKaRa, Beijing, China) according to the manufacturer’s protocol.

qRT-PCR was performed on a CFX Connect Real-Time System (Bio-Rad) with a 20 μL reaction volume, containing 10 μL of TB GREEN ^®^ Premix Ex TaqTM II (Tli RNaseH Plus) (TaKaRa), 0.8 μL of each primer (10 μM), 2 μL of template cDNA, and 6.4 μL sterile distilled water (DNase free). The qRT-PCR protocol was set as follow: 30 s at 95 °C followed by 40 cycles of 5 s at 95 °C and 30 s at 60 °C. A procedure of 0.5 °C increment at 5 s/step from 65 °C to 95 °C was added after for the melt curve analysis. All experiments were operated in triplicate and data were analyzed by the CFX Manager™ Software v3.1 (Bio-Rad, Hercules, California, USA). The relative gene expression level was calculated based on the 2^−ΔΔCT^ method [32] and the actin gene was set as reference.

### 4.7. Statistical Analysis

All the experiments above were conducted in at least triplets to guarantee the reproducibility. Statistical analysis was carried out by using GraphPad Prism 9.0 and Microsoft Excel. A one-way analysis of variance (ANOVA) was applied for the determination of the significant differences from the control groups for each experimental condition separately (*p* < 0.05). All the data are presented in the form as means value (*n* = 3) ± the standard deviation.

## 5. Conclusions

In this study, the *C. zofingiensis* mutant *Cz-pkg* was generated and characterized. Under 30 g L^−1^ glucose inducement, *Cz-pkg* consisted of high amount of lutein and stayed green, while WT accumulated astaxanthin with red phenotype. *Cz-pkg* consists of a dysfunctional PKG, leading to not shutting off its photosystem and staying green, with higher biomass and lutein production under mixotrophy. Specifically, coupled with 4.0% CO_2_ aeration, the mixotrophic *Cz-pkg* with 5 g L^−1^ glucose produced lutein 31.93 ± 1.91 mg L^−1^ with a productivity of 10.57 ± 0.73 mg L^−1^ day^−1^. This study demonstrated that Cz-*pkg* could serve as a promising strain for lutein production.

## Figures and Tables

**Table 1:** Pigment profiles of *Cz-pkg* under different cultivation conditions in autotrophy and mixotrophy. Data in the table were presented in the form of means (*n* = 3) ± the standard deviation.

Cultivation Conditions	Pigment Composition (mg g^−1^ DW)			
	Total Carotenoids	Lutein	Chl a	Chl b
*Autotrophy*				
flasks	5.32 ± 0.31	4.63 ± 0.22	10.63 ± 0.97	2.43 ± 0.30
Bubble tubes + Air	5.28 ± 0.22	4.40 ± 0.37	11.06 ± 1.01	3.41 ± 0.11
Bubble tubes + 2.5% CO_2_	7.12 ± 0.80	5.70 ± 0.20	23.44 ± 2.05	7.56 ± 1.08
Bubble tubes + 4.0% CO_2_	8.40 ± 0.81	7.73 ± 0.52	25.50 ± 2.44	6.86 ± 0.41
Bubble tubes + 5.0% CO_2_	3.78 ± 0.61	3.06 ± 0.19	10.38 ± 1.20	2.25 ± 0.06
*Mixotrophy*				
flasks	5.94 ± 0.33	3.97 ± 0.33	11.37 ± 0.24	2.18 ± 0.50
Bubble tubes + Air	5.83 ± 0.34	4.47 ± 0.10	12.94 ± 0.41	3.67 ± 0.23
Bubble tubes + 2.5% CO_2_	6.72 ± 0.35	4.71 ± 0.25	17.83 ± 1.00	5.02 ± 0.14
Bubble tubes + 4.0% CO_2_	7.82 ± 0.49	6.28 ± 0.57	20.80 ± 1.32	6.93 ± 0.20
Bubble tubes + 5.0% CO_2_	5.67 ± 0.25	2.52 ± 0.28	15.97 ± 1.30	3.46 ± 0.51

**Table 2:** Time-course of lutein contents (mg g^−1^ DW) and the maximal productivity (mg L^−1^ day^−1^) of *Cz-pkg* with different CO_2_ concentration aeration. Data in the table were presented in the form of means (*n* = 3) ± the standard deviation.

Conditions	Lutein Contents (mg g^−1^ DW)				*Pmax* (mg L^−1^ day^−1^)
	Day 2	Day 4	Day 6	Day 8	
Auto + 2.5% CO_2_	2.16 ± 0.06	2.68 ± 0.14	3.25 ± 0.33	5.70 ± 0.35	5.45 ± 0.52
Auto + 4.0% CO_2_	2.50 ± 0.25	2.77 ± 0.08	3.81 ± 0.40	7.73 ± 0.52	8.80 ± 0.60
Mixo + 2.5% CO_2_	2.02 ± 0.13	2.40 ± 0.22	2.98 ± 0.21	4.71 ± 0.25	5.61 ± 0.44
Mixo + 4.0% CO_2_	1.98 ± 0.16	2.85 ± 0.16	3.45 ± 0.17	6.28 ± 0.57	10.57 ± 0.73

Auto, autotrophy; Mixo, mixotrophy with 5 g L^−1^ glucose; *P_max_*, the maximal productivity of lutein.

**Table 3:** Natural sources and several microalgae species potential for lutein production. - represents no related data.

Sources	Lutein Content	Productivity (mg L^−1^ day^−1^)	References
*Tagetes erecta* (Marigold flower)	0.17–5.70 mg g^−1^	-	[3]
Chicken egg yolk	16.22 μg g^−1^	-	[25]
*Brassica oleracea* (Broccoli)	39 μg g^−1^	-	[25]
*Tetracystis intermedium*	3.5 mg g^−1^	-	[25]
*Chlorella sorokiniana* FZU60	11.22 mg g^−1^	8.25	[1]
*Chlorella vulgaris* CS-41	9.0 mg g^−1^	1.56	[1]
*Chlorella* sp. GY-H4	8.9 mg g^−1^	10.50	[1]
*Chlorella sorokiniana* MB-1-M12	7.39 mg g^−1^	3.43	[19]
*Chlorella minutissima* MCC-27	7.05 mg g^−1^	6.34	[19]
*Chlorella vulgaris*	5–9 mg g^−1^	1.60	[19]
*Chlorella sorokiniana* AK-1	4.56 mg g^−1^	3.56	[19]
*Chromochloris zofingiensis* WT	3.07 mg g^−1^	1.24	This study
*Cz-pkg*	7.73 mg g^−1^	10.57	This study

## Data Availability

Not applicable.

## Supplementary Materials

The following are available online at https://www.mdpi.com/article/10.3390/md20030194/s1, Figure S1: The sequence alignment of *PKG* gene of WT (the upper) and *Cz-pkg* and the peptide sequence of PKG in WT, Figure S2: Biomass concentration and the specific growth rate of *Cz-pkg* under cultivation with different glucose concentrations. Table S1: Primers for qRT-PCR analysis. Table S2: Fold change of transcriptional differences of genes related to photosynthetic systems, chlorophyll synthesis and degradation.
