# Supplementary material for: Developing a Chromochloris zofingiensis Mutant for Enhanced Production of Lutein under CO2 Aeration
Source: Mar Drugs. 2022 Mar 7;20(3):194. doi: 10.3390/md20030194 (PMC8950978; doi:10.3390/md20030194)
Supplement: Supplementary file 1 [file marinedrugs-20-00194-s001.zip › Supplementary data 2.pdf]

Supplementary data

Developing a *Chromochloris zofingiensis* mutant for enhanced production of lutein under CO_2 aeration

Yuanyuan Ren [1,2,3], Jinquan Deng [2,3], Yan Lin [2,3], Junchao Huang [2,3,*] and Feng Chen [2,3,*]

1 Institute for Food and Bioresource Engineering, College of Engineering, Peking University, Beijing, 100871, China; 1701111648@pku.edu.cn (Y.R.)

2 Shenzhen Key Laboratory of Marine Microbiome Engineering, Institute for Advanced Study, Shenzhen University, Shenzhen 518060, China; 1900392002@email.szu.edu.cn (J.D.); yanhely@163.com (Y.L.);

3 Institute for Innovative Development of Food Industry, Shenzhen University, Shenzhen 518060, China;

* Correspondence: huangjc65@szu.edu.cn (J.H.) ; sfchen@szu.edu.cn (F.C.)

```
             *         *         *         *         *         *         *         *         *         *
801 TGTTGGAGCGCCGCAAGGGCATCAACATGCGTGTAAATGTGAAGGAGACCGGGGATGTTTTTGGTGAGGTGTCCCTCATGTACAACTGCCCCCGCACAGC 900
    |||#||||||||||||||||||||||||||||||||||||||||||||||||||||||||||||||||||||||||||||||||||||||||||||||||
801 TGTAGGAGCGCCGCAAGGGCATCAACATGCGTGTAAATGTGAAGGAGACCGGGGATGTTTTTGGTGAGGTGTCCCTCATGTACAACTGCCCCCGCACAGC 900
             *         *         *         *         *         *         *         *         *         *
```

MGNSHSQEQSEPSSPPLESEGHGIGRVQAVTGAKAAEPRAKPRGPKANVADY
PRIQGALGKLLLFNRLNKDAQLKIVENMWERSVTAGEILIQEGEVGMAASELY
VVRDGKFEV**L**ERRKGINMRVNVKETGDVFGEVSLMYNCPRTATVAATTDASV
WVLEREVFRKYIQEAAEGEVGQIQLFLNSVPLLNPLTVEAKLKLVDAFSEETF
PTGATIIREGDPGDKFYIIKGGEAVVTQGGKEVNRLFKADFFGERALLRSEPRV
ATVAAVQETVCLVLDRDTFVEILGPFDKVMQEAKSEEASRQRVALFKPRGSAA
AAARPRAPVHIKVKNRSSGYMTVTATGHMDEVQELAQGGTKMGAVDTAPN
GSLLSELMIVEADLLGEGAFSRVSEVTEETTKRTFALKRMTKTAALQCPEHVY
CEQHISKNTANAFCIRQYASFKDQFHLYFLFDLMAGGDLMDVLVAEAKIIKHP
VPQKGSLRQGCLAPKVKMWQGMEEDMAKFYVASVVLALEYLHDNGIVYRD
LKPENVLIDGQGYAKLGDFGFAKQIDVGGRTYTFCGTPGYVAPENVLGRGYN
QSVDWWTLGVLLYVLLTARQPFSSPKTHDPMEVMRRIVDDRWTVKYPPYMS
AAGRDLIQRLLERKPAKRLGMLQGRAADIKNHKWFADFDWAALSARRMDPP
RRPRRTDHAK RKQELEDAHKGDPVEPTMTPEEHAECEKVFADF

Supplemental figure S1. The sequence alignment of PKG gene of WT (the upper) and *Cz-pkg* and the peptide sequence of PKG in WT. The red marker indicates the point mutation (T804A). The highlighted L represents the leucine from the codon UUG.

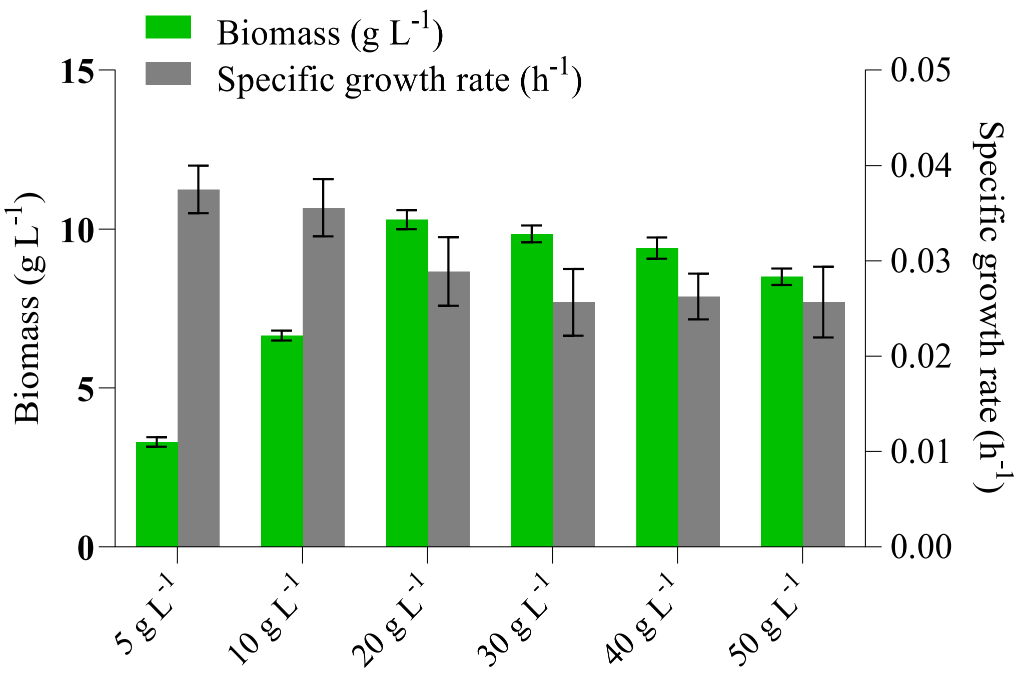

Supplemental figure S2. Biomass concentration and the specific growth rate of *Cz-pkg* under cultivation with different glucose concentrations. Data were presented in the form of mean ± the standard deviation (n = 3).
